# Exogenous Melatonin Improves Plant Iron Deficiency Tolerance via Increased Accumulation of Polyamine-Mediated Nitric Oxide

**DOI:** 10.3390/ijms17111777

**Published:** 2016-10-25

**Authors:** Cheng Zhou, Zhi Liu, Lin Zhu, Zhongyou Ma, Jianfei Wang, Jian Zhu

**Affiliations:** 1 School of Life Science and Technology, Tongji University, Shanghai 200092, China; czhou1224@hotmail.com (C.Z.); liuzhikakashi@163.com (Z.L.); putaojiuvsduyao@126.com (L.Z.); 2 Key Laboratory of Bio-organic Fertilizer Creation, Ministry of Agriculture, Anhui Science and Technology University, Bengbu 233100, China; mazy@ahstu.edu.cn

**Keywords:** melatonin, iron deficiency, polyamine, nitric oxide (NO), iron remobilization

## Abstract

Melatonin has recently been demonstrated to play important roles in the regulation of plant growth, development, and abiotic and biotic stress responses. However, the possible involvement of melatonin in Fe deficiency responses and the underlying mechanisms remained elusive in *Arabidopsis thaliana*. In this study, Fe deficiency quickly induced melatonin synthesis in *Arabidopsis* plants. Exogenous melatonin significantly increased the soluble Fe content of shoots and roots, and decreased the levels of root cell wall Fe bound to pectin and hemicellulose, thus alleviating Fe deficiency-induced chlorosis. Intriguingly, melatonin treatments induced a significant increase of nitric oxide (NO) accumulation in roots of Fe-deficient plants, but not in those of polyamine-deficient (*adc2-1* and d-arginine-treated) plants. Moreover, the melatonin-alleviated leaf chlorosis was blocked in the polyamine- and NO-deficient (*nia1nia2noa1* and c-PTIO-treated) plants, and the melatonin-induced Fe remobilization was largely inhibited. In addition, the expression of some Fe acquisition-related genes, including *FIT1*, *FRO2*, and *IRT1* were significantly up-regulated by melatonin treatments, whereas the enhanced expression of these genes was obviously suppressed in the polyamine- and NO-deficient plants. Collectively, our results provide evidence to support the view that melatonin can increase the tolerance of plants to Fe deficiency in a process dependent on the polyamine-induced NO production under Fe-deficient conditions.

## 1. Introduction

Since the first identification of melatonin (*N*-acetyl-5-methoxytryptamine) from the borine pineal gland [1], considerable efforts have been made to unravel its roles in living organisms, including animals and plants. In animals, melatonin is involved in regulating diverse physiological processes, such as biorhythms, seasonal reproduction, antioxidant functions, and immune stimulation [2,3,4]. Recently, much attention has also been attracted to study the functions of melatonin in plants; it acts as a crucial messenger molecule to regulate lateral root formation [5], flowering [6], fruit ripening [7], and leaf senescence [8]. Apart from its roles in plant growth and development, melatonin can improve the tolerance of plants to adverse environments [9,10,11,12,13].

As sessile organisms, plants are frequently subjected to various biotic or abiotic stresses. Meanwhile, a series of adaptive strategies has been evolved to counteract deleterious effects imposed by stressful factors. It has recently been reported that melatonin can increase the resistance of plants to biotic stress via the salicylic acid (SA)- and nitric oxide (NO)-mediated signaling pathways [14,15]. Furthermore, a large number of publications has documented that melatonin positively participates in the alleviation of plant injury by activating endogenous defense systems against various abiotic stresses, such as UV light, cold, heat, drought, and oxidative stress [16,17,18,19,20,21,22], suggesting that melatonin functions as a novel regulator of abiotic stress responses in plants. However, whether melatonin is involved in the regulation of plant iron (Fe) deficiency responses and the underlying mechanisms are poorly known to date.

Fe deficiency is one of the widest-ranging abiotic stresses that constraints plant growth and yield. To adapt low Fe conditions, plants have developed multiple strategies to take up and translocate Fe [23]. Under Fe deficiency, dicots and non-grass monocots use a reduction-based mechanism, known as strategy I, to enhance Fe solubility and absorption. Strategy I has been well characterized by three main processes: (i) extrusion of protons into the rhizosphere for increasing Fe solubility via H-ATPase (AHA2) [24]; (ii) reduction of ferric iron (Fe^3+^) to ferrous iron (Fe^2+^) by the plasma membrane-bound ferric chelate reductase (FRO2) [25]; and (iii) improvement of Fe uptake by activation of the divalent metal transporter (IRT1) [26]. These physiological processes are finely regulated by limited or sufficient Fe conditions at the molecular level. Fe starvation induces the expression of *AtFRO2*, encoding the ferric-chelate reductase, in *Arabidopsis* plants [25]. Similarly, the expression of the *Arabidopsis IRT1*, encoding the high-affinity Fe transporter, is up-regulated in response to the low Fe supply [27]. The basic helix-loop-helix (bHLH) transcription factor *FIT1* has been demonstrated to regulate the strategy I Fe-deficiency responses and Fe acquisition in plant roots via induction of *IRT1* and *FRO2*. Furthermore, the expression of *FIT1* is transcriptionally controlled by Fe deprivation [28].

Numerous signaling molecules are involved in regulating plant Fe deficiency responses, including nitric oxide (NO) [29,30,31], hydrogen sulfide (H_2_S) [32], carbon monoxide (CO) [33], and carbon dioxide (CO_2_) [34]. NO has been demonstrated to play an important role in controlling Fe uptake and homeostasis [29,30,31]. Disrupted generation of cellular NO affects plant Fe-deficiency responses regulated by the *FIT1* in *Arabidopsis* plants [31]. Interestingly, exogenous polyamines, including putrescine (Put), spermidine (Spd), and spermine (Spm), can rapidly induce a significant increase of NO production in *Arabidopsis* plants [35], suggesting that NO is a key intermediate of polyamine-mediated signaling pathways. More recently, exogenous Put induces the accumulation of NO, thereby activating plant Fe deficiency responses and promoting Fe remobilization from the cell wall [36]. The cell wall is the major reservoir for root apoplastic Fe, and over 75% of Fe is stored in the cell wall of plant roots [37]. Although root apoplastic Fe is not easy to reutilize, accumulating evidence has indicated that Fe retained in the cell wall can be remobilized under Fe deficiency [38,39]. It has been previously shown that nitrosyl-Fe complexes can be formed in plants with endogenous ligands, NO, and Fe [40]. The formation of nitrosyl-Fe complexes not only increases Fe mobilization but also efficiently delivers NO to different plant tissues. Thus, NO can act as a signaling molecule to regulate plant Fe deficiency responses, and serves as a chelator of Fe. A recent study has reported that exogenous application of melatonin obviously increases endogenous NO levels in *Arabidopsis* plants [15], although the detailed mechanism behind melatonin regulates cellular NO biosynthesis remains unclear. Shi et al. [12] have reported that exogenous melatonin affects polyamine metabolic pathways in oxidative stress-treated Bermuda grass. Melatonin can alleviate cold-induced apoptosis in carrot suspension cells by increasing the content of Put and Spd [41]. Additionally, melatonin treatments enhance the tolerance of harvested peach fruits to chilling stress, which is associated with the increased polyamine content [42]. These findings indicate that melatonin regulates plant abiotic stress responses, possibly involving polyamine-mediated NO accumulation.

In the present study, Fe deficiency caused the increased melatonin levels in *Arabidopsis* plants. Furthermore, melatonin treatments markedly alleviated plant Fe deficiency-induced chlorosis, but not in the polyamine- and NO-deficient plants. This study indicated that melatonin regulated Fe deficiency responses and promoted Fe remobilization from the cell wall via induction of polyamine-mediated NO accumulation.

## 2. Results

### 2.1. Effects of Fe Deficiency on the Content of Melatonin and Polyamine in Arabidopsis

**Figure 1:**
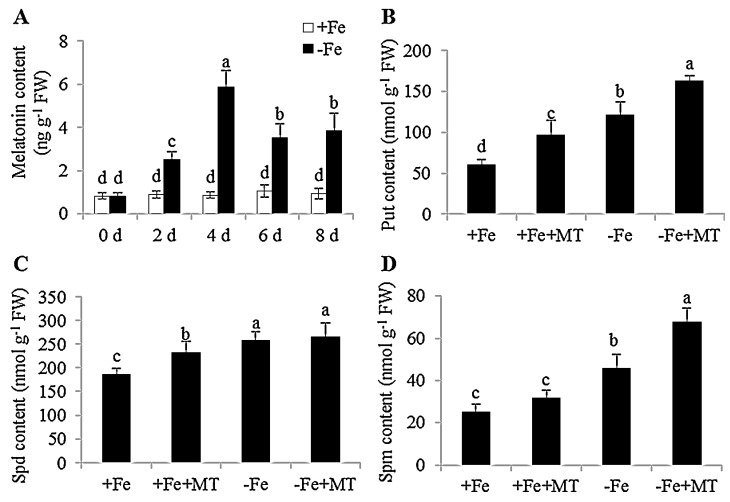
Changes of endogenous melatonin and polyamine content in response to Fe deficiency, and the effects of exogenous melatonin on cellular polyamine levels in *Arabidopsis* plants. Seven-day-old seedlings were placed in 1/2 MS liquid medium with or without the presence of 50 μM Fe for the indicated time, then these plants were taken to measure melatonin content (**A**). In addition, seve-day-old seedlings were treated with or without 5 μM melatonin under −Fe and +Fe conditions for 8 days. These plants were then used to determine cellular polyamine, including Put (**B**), Spd (**C**), and Spm (**D**). Each bar is the mean ± SE of at least three replicates, and different lowercase letters above the bars indicating significant differences using a Tukey’s test at *p* < 0.05.

To examine the effects of Fe deficiency on the content of endogenous melatonin in plants, seven-day-old *Arabidopsis* plants were exposed to Fe-deficient (−Fe) conditions for eight days (d). Fe deficiency rapidly induced the biosynthesis of melatonin in the *Arabidopsis* plants (Figure 1A). After 4 days of exposure to Fe deficiency, the melatonin levels in plants were elevated about six-fold as compared to non-treated plants, and then dropped gradually with the following treatments. Concomitantly, the content of polyamines, including Put, Spd, and Spm, was also significantly increased after 8 days of exposure to Fe deficiency (Figure 1B–D). Interestingly, exogenous application of 5 μM melatonin induced the increment of cellular polyamine levels in plants under Fe-sufficient (+Fe) and −Fe conditions, while the content of polyamine was markedly higher in the melatonin-treated plants grown under −Fe conditions than under +Fe conditions. Moreover, melatonin treatments markedly up-regulated the expression levels of two arginine decarboxylase (ADC) genes, *ADC1* and *ADC2*, in *Arabidopsis* plants. Consistently, the activities of ADC were significantly enhanced in the melatonin-treated plants compared with non-treated plants under +Fe and −Fe conditions (Figure S1). Recently, exogenous Put has been shown to improve the tolerance of *Arabidopsis* plants to Fe deficiency [36]. Thus, the melatonin-induced increase of polyamine levels indicated the possible involvement of melatonin in Fe deficiency responses of plants.

### 2.2. Involvement of Polyamine in Plant Fe Deficiency Tolerance Conferred by Melatonin

**Figure 2:**
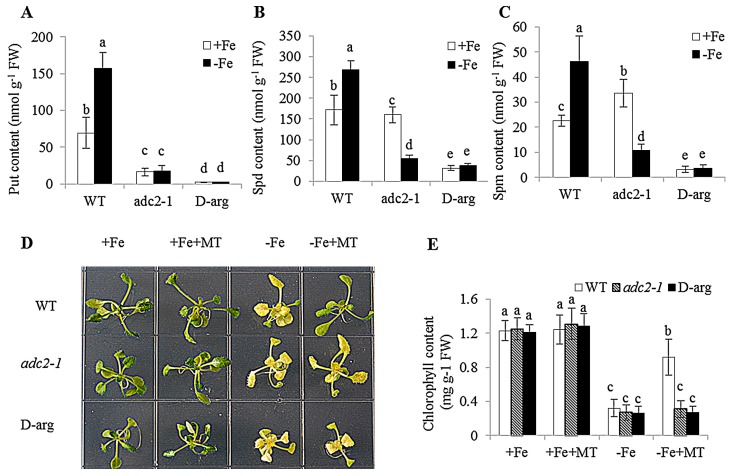
The effect of exogenous melatonin on Fe deficiency symptoms in the WT, *adc2-1*, and d-arg-treated *Arabidopsis* plants. Seven-day-old seedlings were grown under +Fe (50 μM Fe) and −Fe (0 μM Fe) conditions for 8 days, then these plants were sampled to measure the content of endogenous polyamine, including Put (**A**), Spd (**B**), and Spm (**C**). Additionally, seven-day-old seedlings were grown under +Fe and −Fe conditions with or without the presence of 5 μM melatonin for 8 days, respectively. Then, these plants were used to analyze the growth phenotype (**D**) and chlorophyll content (**E**). Each bar is the mean ± SE of at least three replicates, and different lowercase letters above the bars indicate significant differences using Tukey’s test at *p* < 0.05.

**Figure 3:**
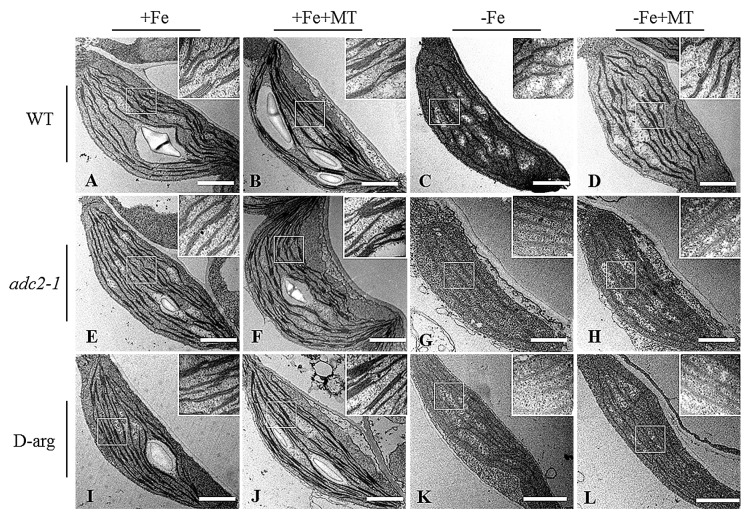
Transmission electron micrographic (TEM) analyses of the effect of exogenous melatonin on the chloroplast ultrastructure in the WT (**A**–**D**), *adc2-1* (**E**–**H**), and d-arg-treated (**I**–**L**) *Arabidopsis* plants. Seven-day-old seedlings were treated with or without 5 μM melatonin in the presence or absence of 50 μM Fe for 8 days. Then the leaves from these plants were sampled to analyze the chloroplast ultrastructure: (**A**,**E**,**I**), +Fe; (**B**,**F**,**J**), +Fe + MT; (**C**,**G**,**K**), −Fe; (**D**,**H**,**L**), −Fe + MT; Scale bar = 1 μm.

To determine whether the mechanisms behind melatonin improved Fe deficiency tolerance was associated with the metabolic pathways of polyamine, the *adc2-1* mutant, in which the Put level is reduced by about 75% of wild-type (WT) *Arabidopsis* plants [43], was used. Approximately a 70%–90% reduction of polyamine content was found in the *adc2-1* mutant under −Fe conditions compared with WT plants (Figure 2A–C). There was no phenotypic difference between WT and *adc2-1* under +Fe or +Fe + MT treatments. However, under Fe deficiency, leaf chlorosis appeared more apparent in the *adc2-1* than WT plants, but was not remarkably relieved by melatonin treatments (Figure 2D). Moreover, d-arginine (d-arg), an inhibitor of ADC, was used to treat WT plants. Cellular polyamine levels were significantly lower in the d-arg-treated plants than the WT plants (Figure 2A–C). Additionally, the d-arg-treated plants exhibited a more severe Fe-deficiency phenotype under −Fe or −Fe + MT treatments compared with the WT plants (Figure 2D). In addition, the chlorophyll content of WT plants was much similar to that of *adc2-1* or d-arg-treated plants under +Fe or +Fe + MT treatments (Figure 2E). Under Fe deficiency, melatonin treatments could not pronouncedly increase the chlorophyll content of *adc2-1* or d-arg-treated plants, in accordance with the phenotype observed. Transmission electron micrographic (TEM) analyses further revealed that exogenous melatonin promoted chloroplast development with an increased number of normal grana stacking in the WT plants, but no observable effects were found in the *adc2-1* and d-arg-treated plants (Figure 3).

**Figure 4:**
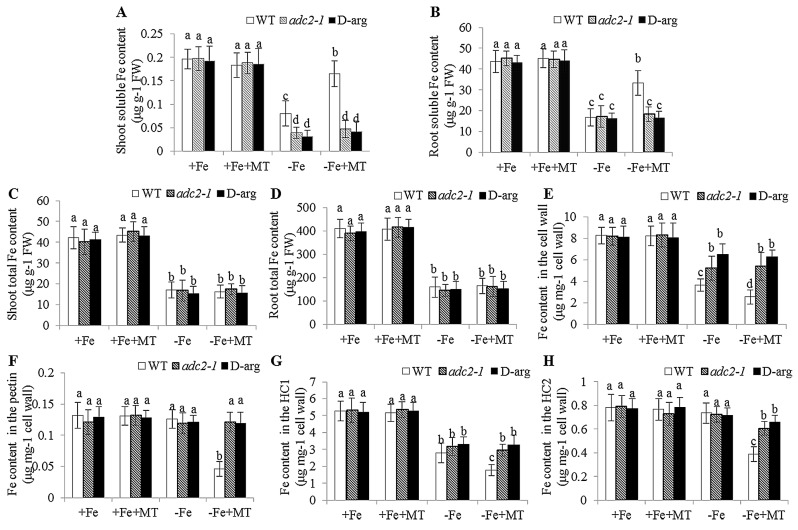
The effect of exogenous melatonin on Fe content in the WT, *adc2-1* and d-arg-treated *Arabidopsis* plants. Seven-day-old seedlings were treated with or without 5 μM melatonin in the presence or absence of 50 μM Fe for 8 days. Then these plants were taken to measure the soluble Fe content of shoots (**A**) and roots (**B**), the total Fe content of shoots (**C**) and roots (**D**), and to measure the Fe content in root cell wall components: total cell wall (**E**), pectin (**F**), HC1 (**G**), and HC2 (**H**) fractions. Each bar is the mean ± SE of at least three replicates, and different lowercase letters above the bars indicate significant difference using Tukey’s test at *p* < 0.05.

Soluble Fe content was further examined in shoots and roots of WT, *adc2-1*, and d-arg-treated plants. The soluble Fe content of shoots was less in the *adc2-1* and d-arg-treated plants than in the WT plants under −Fe conditions, but there were no pronounced differences in the soluble Fe content of the roots (Figure 4A,B) or total Fe content (Figure 4C,D) of the shoots and roots. Moreover, melatonin treatments increased the soluble Fe content of the shoots and roots in the WT plants under −Fe treatments, but not in both the *adc2-1* and d-arg-treated plants. Interestingly, there was no available Fe in the −Fe medium, which raised the question of the source of soluble Fe in plants. Since the cell wall plays pivotal roles in Fe reutilization in *Arabidopsis* plants during Fe deficiency [39], we wanted to know how melatonin treatments enhanced the release of cell wall-retained Fe under −Fe conditions. In this study, the cell wall of WT roots contained less bound Fe than *adc2-1* and d-arg-treated roots under −Fe treatments, and exogenous melatonin caused a further decrease in the WT roots, but not in the *adc2-1* and d-arg-treated roots (Figure 4E). In addition, Fe retained in the pectin was decreased only under −Fe + MT treatments (Figure 4F), but the HC1 fraction had a similar pattern as that of the cell wall (Figure 4G). The Fe in the HC2 fraction was decreased under −Fe + MT treatments compared with those under −Fe treatments (Figure 4H), but no significant difference was observed under other treatment conditions. These results indicated that the melatonin-induced increase of soluble Fe might be attributed to efficient remobilization of cell wall Fe, which was associated with the polyamine levels.

### 2.3. Exogenous Melatonin Increased the Polyamine-Mediated NO Production in Arabidopsis Roots

**Figure 5:**
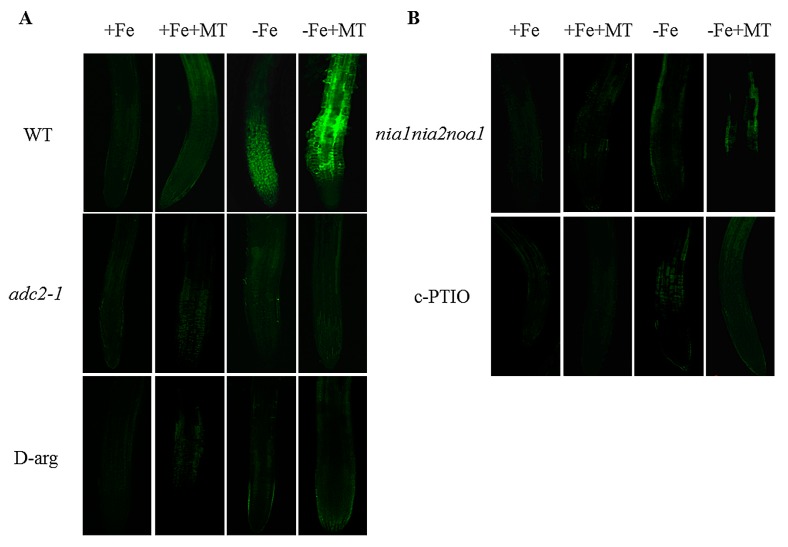
The effect of melatonin on the NO accumulation in roots of polyamine- and NO-deficient *Arabidopsis* plants. Seven-day-old seedlings were treated with or without 5 μM melatonin in the presence or absence of 50 μM Fe for 8 days. Then, NO-associated fluorescence was detected in the roots of polyamine-deficient (*adc2-1* and d-arg-treated) (**A**) and NO-deficient (*nia1nia2noa1* and c-PTIO-treated) plants (**B**).

Previously, polyamine has been shown to rapidly induce NO accumulation in plants [35]. In this study, exogenous melatonin induced a significant increase of polyamine content in *Arabidopsis* plants. Therefore, we speculated that exogenous melatonin affected Fe deficiency responses via induction of polyamine-mediated NO accumulation. Here, a cell-permeable NO-sensitive diaminofluorescein diacetate (DAF-FM DA) was used to detect NO accumulation in *Arabidopsis* roots. As shown in Figure 5A, the level of NO-associated fluorescence was extremely low in the roots of non-treated WT plants, but significantly increased after 16 h of exposure to melatonin under +Fe conditions. When plants were exposed to −Fe conditions, the level of NO was remarkably increased, and melatonin treatments led to an even further elevation of the NO accumulation in roots. Additionally, the level of NO-associated fluorescence was also analyzed in the roots of *adc2-1* and d-arg-treated plants. The NO-associated fluorescence was markedly reduced in the roots of *adc2-1* and d-arg-treated plants. Moreover, the roots of *adc2-1* plants accumulated a lower NO level than those of WT plants under the same conditions. We found that the roots of d-arg-treated plants could not sustain melatonin-induced increases of NO accumulation, which possibly resulted from the block of polyamine-mediated NO synthesis. Furthermore, we analyzed the effects of exogenous melatonin on a triple *nia1nia2noa1* mutant that is impaired in nitrate reductase (NIA)- and nitric oxide-associated1 (NOA1)-mediated NO biosynthetic pathways [44]. As shown in Figure 5B, low levels of NO were detected in the roots of both the *nia1nia2noa1* under +Fe or +Fe + MT treatments, and exogenous melatonin could not increase the accumulation of NO under −Fe treatments. Similar results were shown in which melatonin treatments could not enhance the NO accumulation in plants treated with 2-(4-carboxyphenyl)-4,4,5,5-tetramethylimidazoline-1-1-oxy-3-oxide (PTIO), a NO-specific scavenger PTIO.

### 2.4. NO Is Involved in the Regulation of Plant Fe Deficiency Responses by Melatonin

**Figure 6:**
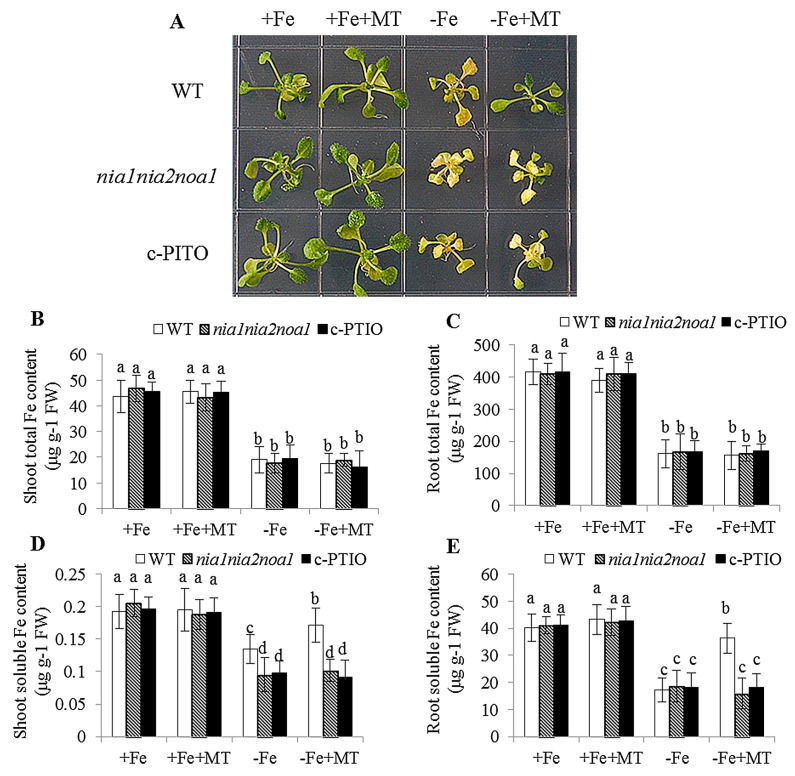
The effect of exogenous melatonin on Fe deficiency symptoms in the WT, *nia1nia2noa1*, and c-PTIO-treated *Arabidopsis* plants. Seven-day-old seedlings were treated with or without 5 μM melatonin in the presence or absence of 50 μM Fe for 8 days. Then, these plants were used to analyze the growth phenotype (**A**), total Fe content of shoots (**B**) and roots (**C**), and soluble Fe content of shoots (**D**) and roots (**E**). Each bar is the mean ± SE of at least three replicates, and different lowercase letters above the bars indicate significant differences using Tukey’s test at *p* < 0.05.

**Figure 7:**
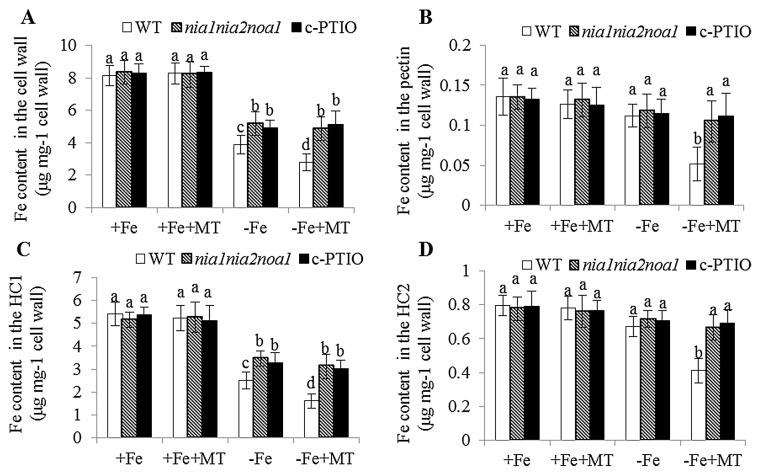
The effect of exogenous melatonin on Fe content in root cell wall components in the WT, *nia1nia2noa1* and c-PTIO-treated *Arabidopsis* plants. Seven-day-old seedlings were grown in −Fe (0 μM Fe) or +Fe (50 μM Fe) medium without or with the presence of 5 μM melatonin (+MT) for 8 days. Then, these plants were taken to measure the Fe content in the root total cell wall (**A**), pectin (**B**), HC1 (**C**), and HC2 (**D**) fractions. Each bar is the mean ± SE of at least three replicates, and different lowercase letters above the bars indicate significant difference using Tukey’s test at *p* < 0.05.

**Figure 8:**
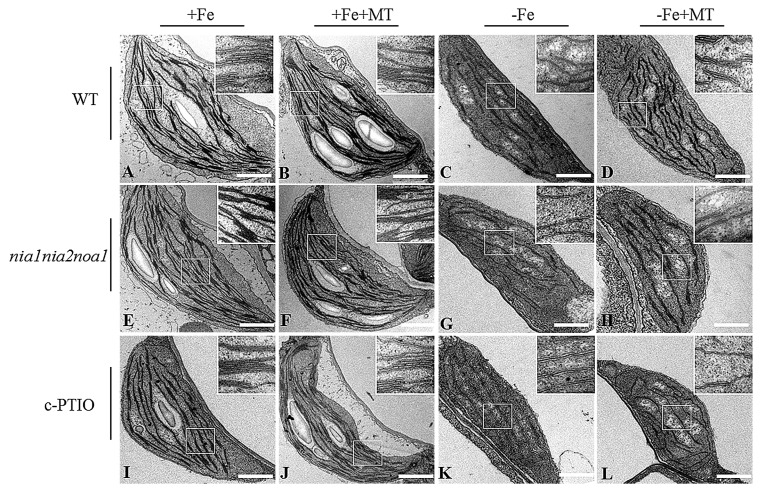
The effect of exogenous melatonin on chloroplast ultrastructure in the WT (**A**–**D**), *nia1nia2noa1* (**E**–**H**), and c-PTIO-treated (**I**–**L**) *Arabidopsis* plants. Seven-day-old seedlings were treated with or without 5 μM melatonin in the presence or absence of 50 μM Fe for 8 days. Then the leaves from these plants were sampled for transmission electron micrographic analyses of the chloroplast ultrastructure: (**A**,**E**,**I**), +Fe; (**B**,**F**,**J**), +Fe + MT; (**C**,**G**,**K**), −Fe; (**D**,**H**,**L**), −Fe + MT; Scale bar = 1 μm.

Phenotypic analyses revealed that leaf chlorosis appeared more pronounced in the NO-deficient plants, including *nia1nia2noa1* and c-PTIO-treated plants under −Fe conditions, relative to WT plants, and could not be alleviated by exogenous melatonin (Figure 6A). Furthermore, there was no difference in the soluble Fe content of roots or total Fe content of shoots and roots among WT, *nia1nia2noa1*, and c-PTIO-treated plants under the same conditions (Figure 6B,C). Under −Fe conditions, exogenous melatonin significantly increased soluble Fe content of shoots and roots in the WT plants, but not in both *nia1nia2noa1* and c-PTIO-treated plants (Figure 6D,E). We also measured Fe content of the root cell wall. As shown in Figure 7, exogenous melatonin led to greater reduction in the Fe content of total cell wall, pectin, HC1, and HC2 fractions in the WT plants under −Fe conditions than those in both the *nia1nia2noa1* and c-PTIO-treated plants. In addition, the results of TEM analyses revealed that exogenous melatonin caused a marked increase of normal grana stacking in the WT plants versus the *nia1nia2noa1* and c-PTIO-treated plants (Figure 8).

**Figure 9:**
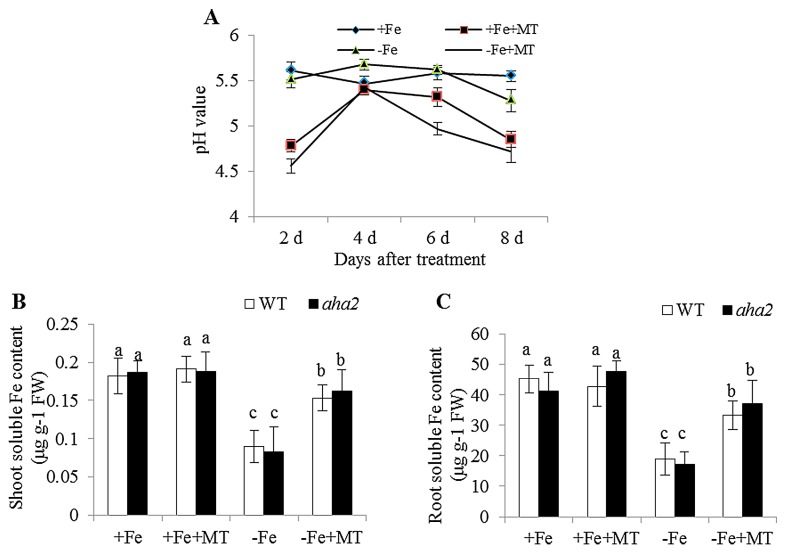
The effect of exogenous melatonin on the pH values of *Arabidopsis* plants. Seven-day-old seedlings were grown in −Fe (0 μM Fe) or +Fe (50 μM Fe) medium (pH = 5.6) without or with the presence of 5 μM melatonin (−MT or +MT) for the indicated times for measuring the pH values (**A**). In addition, soluble Fe content of shoots (**B**) and roots (**C**) were determining after seven-day-old WT and *aha2* were placed in −Fe or +Fe medium with or without the presence of 5 μM melatonin for 8 days. Each bar is the mean ± SE of at least three replicates, and different lowercase letters above the bars indicate significant differences using a Tukey’s test at *p* < 0.05.

NO has been demonstrated to regulate plant acclimation to Fe deficiency through activation of *FIT1*, which is essential for high-level induction of some Fe-acquisition genes, including *FRO2* and *IRT1* [28]. Recently, the increased release of protons to the rhizosphere and ferric chelate reductase (FCR) activity has been taken as important hallmarks of Fe deficiency in plants [29]. Here, it was observed that exogenous melatonin resulted in a marked decrease of the pH value in roots (Figure 9A). It has previously been indicated that lower pH is conducive to increasing the availability of Fe for plants, and rhizospheric acidification induced by Fe deficiency is primarily modulated by AHA2 [24]. However, there was no difference in the soluble Fe content of shoots and roots between WT and the *aha2* mutant under −Fe or −Fe + MT treatments (Figure 9B,C), indicating that the effects of melatonin observed here was not attributable to lower pH conditions.

**Figure 10:**
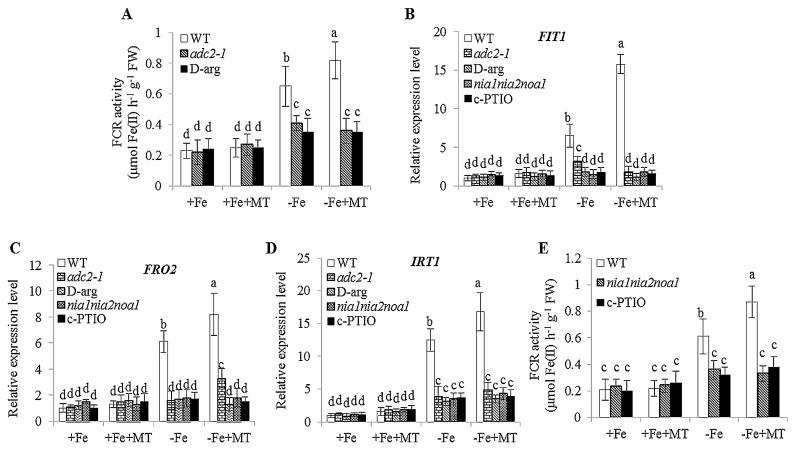
The effect of exogenous melatonin on the FCR activities and the expression of Fe-acquisition-related genes in polyamine- (*adc2-1* and d-arg-treated) and NO-deficient (*nia1nia2noa1* and c-PTIO-treated) *Arabidopsis* plants. Seven-day-old seedlings were treated with or without 5 μM melatonin in the presence or absence of 50 μM Fe for 8 days. Roots from *adc2-1* and d-arg-treated plants were sampled for measuring the FCR activities (**A**). The expression levels of Fe acquisition-related genes, including *FIT1* (**B**), *FRO2* (**C**), and *IRT1* (**D**), were examined after seven-day-old seedlings were placed in the −Fe or +Fe medium with or without the presence of 5 μM melatonin (−MT or +MT) for 8 days. In addition, roots from *nia1nia2noa1* and c-PTIO-treated plants were used to determine the FCR activities (**E**). Each bar is the mean ± SE of at least three replicates, and different lowercase letters above the bars indicate significant differences using Tukey’s test at *p* < 0.05.

Moreover, the FCR activities were remarkably increased under −Fe + MT treatments, but lower activities of FCR were found in the *adc2-1* mutant and d-arg-treated plants than those in the WT plants, and could not be rescued by application of melatonin (Figure 10A). Additionally, Fe deficiency significantly increased the transcription level of *FIT1*, *FRO2*, and *IRT1* in the roots of WT plants, and their expression was further enhanced after melatonin treatments. However, the enhanced expression of these genes was largely inhibited in the *adc2-1* and d-arg-treated plants under −Fe + MT treatments (Figure 10B–D). Similar results were also observed, as the expression levels of *FIT1*, *FRO2*, and *IRT1* were remarkably higher in the WT plants than the *nia1nia2noa1* and c-PTIO-treated plants under −Fe conditions. Additionally, the FCR activities were not significantly enhanced in these NO-deficient plants after exposure to melatonin treatments (Figure 10F). Hence, our results indicated that the ability of melatonin to activate Fe deficiency responses was dependent on the polyamine-induced NO accumulation.

## 3. Discussion

Although several physiological roles of melatonin in plants have been characterized [5,6,7,8,9,10,11,12,13,14,15,16,17,18,19,20,21,22], regulation of plant Fe deficiency responses and Fe homeostasis by melatonin have not been reported. The present study provided evidence that exogenous melatonin conferred improved plant adaptation to Fe deficiency by promoting Fe remobilization in *Arabidopsis* plants. This process was found to be associated with cellular polyamine levels. Furthermore, melatonin treatments could not relieve Fe deficiency-induced chlorosis in the polyamine- and NO-deficient plants. These results indicated that exogenous melatonin increased the resistance of plants to Fe deficiency via induction of polyamine-mediated NO accumulation.

### 3.1. Exogenous Melatonin Enhances the Tolerance of Plants to Fe Deficiency

In higher plants, approximately 90 percent of leaf Fe is located in the chloroplasts [45]. Abundant Fe is essential for maintaining the structural and functional integrity of thylakoid membranes in the chloroplasts [46,47]. Chlorophyll constitutes the major component of the chloroplasts and is positively correlated with leaf Fe status [48]. It has previously been shown that Fe deficiency leads to a reduction of chlorophyll content and degradation of protein components in the chloroplasts which, lastly, disrupts the chloroplast ultrastructure [28,29,30,31,32]. In this study, the *Arabidopsis* seedlings grown under Fe deficiency exhibited serious chlorotic symptoms with yellowing leaves and lower chlorophyll content. Furthermore, TEM analyses of Fe-deficient mesophyll cells showed the chloroplasts with fewer photosynthetic lamellae and grana, displaying disorder of the thylakoid arrangement caused by Fe deficiency. Interestingly, we found that endogenous melatonin content was significantly increased in Fe-deficient *Arabidopsis* plants, indicating the beneficial effects of melatonin on plants exposed to Fe-deficient conditions. As expected, application of melatonin obviously increased the numbers of normal grana stacking and grana lamellae, as well as chlorophyll content in the chloroplasts under Fe deficiency, thereby alleviating leaf chlorosis. These results suggested that melatonin could improve plant Fe nutrition and chloroplast development under −Fe conditions.

### 3.2. The Polyamine Levels Are Responsible for Melatonin-Alleviated Plant Fe Deficiency

How does melatonin regulate the Fe nutrition status in the Fe-deficient *Arabidopsis* plants? Evidence gathered in recent years has indicated that melatonin improves the tolerance of plants to abiotic stress, which relates to the metabolic pathways of polyamine [12,41,42]. Lei et al. [41] have reported that the melatonin-induced increase of polyamine, including Put and Spm, is responsible for mitigating cold-induced apoptosis in carrot suspension cells. Moreover, Cao et al. [42] have reported that melatonin treatment markedly up-regulates the expression of polyamine biosynthesis-related genes, such as *PpADC* and *PpODC*, and results in a significant increase of polyamine levels in harvested peach fruit, which contributes to enhancing the tolerance of harvested peaches to chilling stress. Similar results were found in this study showing exogenous melatonin rapidly induced the polyamine biosynthesis in *Arabidopsis* plants under +Fe and −Fe conditions. Recently, Zhu et al. [36] have indicated that Put plays an important role in the regulation of Fe deficiency responses in *Arabidopsis* plants. Therefore, it seemed reasonable to assume that melatonin conferred the stronger ability of plants to tolerate Fe deficiency, possibly through regulation of polyamine metabolism. Here, we found that the melatonin-induced Fe deficiency tolerance was fully repressed by the ADC inhibitor, d-arg. In concert with this, melatonin treatments could not alleviate Fe deficiency symptoms in the Put-deficient mutant, *adc2-1*, implying the involvement of polyamine in melatonin-regulated Fe deficiency responses.

### 3.3. Melatonin Regulated Plant Fe Deficiency Responses, Which Is Dependent on the Action of NO

Are certain signals downstream of polyamine involved in improving plant Fe nutrition? It has recently been indicated that NO functions as a key signal molecule in the adaptive mechanisms of plants to Fe-deficient conditions [29,30,31]. Ramirez et al. [49] have shown that NO can greatly increase Fe availability in plants and activate Fe-dependent ferritin expression, which alleviates leaf chlorosis and oxidative damage caused by Fe deficiency. NO has also been shown to enhance the activity of FCR by up-regulation of *FRO1* expression in tomato and *Arabidopsis* plants, involving the NO-mediated expression of *FER* or *FIT1* [30,31]. In this study, Fe deficiency led to an increase of the NO levels in the roots, which was positively correlated with up-regulated expression of *FIT1*, *FRO2*, and *IRT1*. Melatonin treatments further caused a greater elevation in the roots, while this induction was largely inhibited by the addition of an NO scavenger, c-PTIO, or in the triple *nia1nia2noa1* mutant. It has previously been reported that polyamine can stimulate the biosynthesis of NO in *Arabidopsis* roots [35]. Furthermore, interruption of NO release has been shown to inhibit certain effects of polyamine on plants [36,50], indicating that NO acts as a downstream transducer of polyamine-mediated signaling pathways. Intriguingly, overproduction of NO can provoke toxic effects in plants [51]. Therefore, a delicate balance of cellular NO levels may be required for the tolerance of plants to various stresses. In this study, melatonin treatments induced the accumulation of NO levels in plant roots, and further alleviated plant Fe deficiency. However, the melatonin-induced increase of NO accumulation did not cause damage to plants under +Fe and −Fe conditions. Thus, the production of NO induced by exogenous melatonin did not exceed a certain threshold value that had adverse physiological consequences.

Does melatonin regulate plant Fe deficiency responses by the polyamine-mediated NO accumulation? Here, we demonstrated that NO acted downstream of polyamine to enhance root FCR activity. When the polyamine levels were significantly decreased in a mutant line (*adc2-1*) and d-arg-treated plants, the NO levels and transcription of Fe-acquisition-related genes, including *FRO2*, *IRT1*, and *FIT1*, were changed accordingly, and these changes were not be reversed by melatonin treatments. Similarly, exogenous melatonin failed to induce the expression of these Fe-acquisition-related genes, and relieve the Fe-deficiency symptoms in both *nia1nia2noa1* and c-PTIO-treated plants. Hence, the polyamine-mediated NO accumulation was responsible for the improved adaptation of plants to Fe deficiency conferred by the application of melatonin. Furthermore, our data revealed that exogenous melatonin obviously increased the soluble Fe content of shoots and roots in *Arabidopsis* plants, but no significant changes in their total Fe content were observed. These similar results were found in the NO-deficient plants, including *nia1nia2noa1* and c-PTIO-treated plants. It has been previously indicated that NO can enhance the root responses to Fe deficiency by activation of *FRO2* and *IRT1* expression regulated by *FIT1* [31]. It was interesting that overexpression of *AtFRO2* [25] or *AtIRT1* [27] in *Arabidopsis* plants did not increase Fe content compared with WT plants, indicating that the increased expression of Fe acquisition-related genes is not sufficient to confer a marked enhancement of Fe levels when plants are grown under conditions of low Fe availability [30]. Therefore, the enhanced ability of melatonin-treated plants to tolerate −Fe conditions may result from an increase in the availability of endogenous iron.

Previous studies have indicated that NO can promote the delivery of Fe between, and within, plant cells through the formation of Fe-nitrosyl complexes [40], thereby increasing the availability of cell wall Fe efficiently. It is well known that the cell wall plays a critical role in the adaptation of plants to short-term Fe-deficient conditions [39]. Reutilization of Fe stored in older leaves and roots has been evolved to facilitate plant survival under limited Fe supply [36,39]. In this study, we found that the decrease in cell wall Fe content was more pronounced in the melatonin-treated plants than non-treated WT plants when plants were grown in the medium without the source of Fe. These results implied that melatonin treatments increased the resistance of plants to Fe deficiency by enhancing remobilization of cell wall Fe. As expected, the melatonin-induced decrease of cell wall Fe or the increase of soluble Fe was not observed in the NO-deficient plants. Thus, increased reutilization of cell wall Fe was dependent on the melatonin-induced NO production.

## 4. Materials and Methods

### 4.1. Plant Materials and Growth Conditions

Wild-type (WT) seeds of *Arabidopsis thaliana* (ecotype Col-0) were used and all of the mutants were generated from this background. Seeds from the *adc2-1* (Salk_026916C), *aha2* (Salk_022010), *nia1nia2* (CS6511), and *noa1-2* (CS2356) mutants were obtained from the *Arabidopsis* Biological Resource Center (ABRC). The *nia1nia2* plants were crossed with the *noa1-2* plants to generate triple *nia1nia2noa1* mutants. The seeds were surface-sterilized and grown in Murashige and Skoog (MS) agar medium for seven days (d) under the conditions of 21 °C with a 16 h/8 h light/dark cycle (130 μmol·m^−2^·s^–1^). Subsequently, these seedlings were transferred to 1/2 MS liquid medium and experienced the following treatments for 8 days: +Fe (1/2 MS liquid medium), +Fe + melatonin (MT) (liquid medium plus 5 μM MT), −Fe (liquid medium without Fe), and −Fe + MT (−Fe liquid medium plus 5 μM MT).

### 4.2. Determination of Endogenous Melatonin and Polyamine Content

Endogenous melatonin was extracted from the *Arabidopsis* plants using the buffer solution (acetone:methanol:water = 89:10:1) according to the method described by Pape and Lüning [52]. After protein precipitation and centrifugation, the content of melatonin was determined using melatonin enzyme-linked immunosorbent assay (ELISA) kit (EK-DSM; Buhlmann Laboratories AG, Schonenbuch, Switzerland) as recently reported by Shi et al. [16]. Additionally, the content of polyamines, including Put, Spd, and Spm, was quantified following the method described by Imai et al. [53]. Approximately 500 mg of *Arabidopsis* plants were homogenized in 5 mL of 5% (*w*/*v*) perchloric acid (PCA). After centrifugation at 12,000× *g* for 10 min, the supernatant was subjected to successive treatments including hydrolysis, dryness, and dansylation. Finally, the extracted polyamines were determined by high performance liquid chromatography (Waters 2695 Alliance; Waters, Milford, MA, USA) with a reverse phase C18 column (250 mm × 4.6 mm, 5 μm particle size). A 1 μL sample was applied and eluted with methanol into the chromatography using methanol-water (70:30, *v*/*v*) as the mobile phase at a flow rate of 0.8 mL/min, and monitored with a Waters™ 996 photodiode array detector at 265 nm.

### 4.3. Measurement of Fe Levels

To analyze total Fe levels, harvested shoots and roots were washed three times with 1.5 mM CaCl_2_ and digested in HNO_3_/HClO_4_ (4:1, *v*/*v*). The content of Fe was determined by inductively-coupled plasma spectrometry (ICP-AES, Perkin Elmer Optimal 2100DV) according to the methods described by Lei et al. [39]. Analyses of water-soluble Fe were performed according to the method described by Cassin et al. [54]. Shoots and roots were ground in liquid nitrogen and extracted with five volumes of deionized water. After centrifugation, the supernatant was collected to measure the concentration of soluble Fe. Cell wall Fe was detected according to the methods recently reported by Lei et al. [39]. Briefly, extraction of crude cell wall was carried out as previously reported by Zhong and Lauchli [55]. Dry cell wall was suspended in 1 mL 2 M HCl for 96 h at room temperature. After centrifugation, the supernatant was collected to measure the content of cell wall Fe. Hemicellulose 1 (HC1), hemicellulose 2 (HC2), and pectin fractions were prepared for determining their total Fe content.

### 4.4. Assays of FCR and ADC Activity

Root FCR activity was quantified according to the method reported by Lei et al. [39], with minor revision. Briefly, the roots were excised from plants and were then immediately immersed in the assay solution containing 0.1 mM ferrous ethylene diamine tetraacetic acid (EDTA-Fe), 0.5 mM CaSO_4_, and 0.2 mM Ferrozine. Subsequently, these roots were placed in a dark room at 21 °C for 3 h. Absorbance of the assay solutions was determined by a spectrophotometer at 562 nm. The concentration of Fe(II) was calculated using an extinction coefficient of 28.6 mM^−1^·cm^−1^. Additionally, the ADC activity was examined according to the method reported by Rossi et al. [56] with minor modifications. The *Arabidopsis* plants were homogenized in extraction buffer (0.5 mM EDTA, 1 mM pyridoxal phosphate (PLP), 1 mM phenylmethanesulfonyl fluoride (PMSF), 10 mM dithiothreitol (DTT), 15 mM KH_2_PO_4_, 20 mM sodium ascorbate, and 85 mM Na_2_HPO_4_, pH 7.5). After centrifugation at 12,000× *g* for 20 min, the supernatant was added into a reaction mixture containing 50 nCi (U-^14^C)-l-arginine, 10 mM l-arginine, and 1 mM PLP. Subsequently, the ^14^CO_2_ released was quantified by densitometry.

### 4.5. Gene Expression Analyses

Total RNA was extracted from root tissues using Trizol reagent (Takara, Dalian, China) and 500 ng of total RNA was used as the template for first-strand cDNA synthesis using a PrimeScript RT reagent kit according to the manufacturer’s instruction (Takara). All RNA samples were digested with DNAase before reverse transcription. Real-time quantitative RT-PCR (qRT-PCR) was conducted was conducted with an ABI 7300 real-time PCR system (Applied Biosystems, Carlsbad, CA, USA) with the following reaction conditions: 30 s at 94 °C; 15 s at 95 °C; and 30 s at 60 °C for 40 cycles. The housekeeping gene *actin 2* was used as used as an internal control to normalize relative transcript levels of targeted genes. Each experiment was conducted three times with different cDNA samples from independent biological replicates. The primers used in this study including *FIT1*, *FRO2*, *IRT1*, and *actin2* were shown in Table S1.

### 4.6. NO Detection by Confocal Microscopy

In vivo localization of NO in *Arabidopsis* roots was detected using 4-amino-5-methylamino-2′,7′-difluorofluorescein diacetate DAF-FM DA probes as described by Zhu et al. [36]. To analyze the levels of NO, the *Arabidopsis* roots were loaded with 2.0 μM DAF-FM DA in the dark for 20 min, and then washed at least three times in phosphate-buffered saline (PBS) (pH 7.2) solution. NO-associated fluorescence was detected using a Leica SP2-AOBS confocal microscope (Leica, Wetzlar, Germany) with an excitation filter of 490 nm and an emission filter of 520 nm.

### 4.7. Statistical Analyses

Each experiment was performed with at least three biological repeats. Each bar is the mean ± SE of at least three replicates. Different letters above the bars indicate significant differences and *p* < 0.05 was considered to represent statistical significance using the analysis of Tukey’s test.

## 5. Conclusions

**Figure 11:**
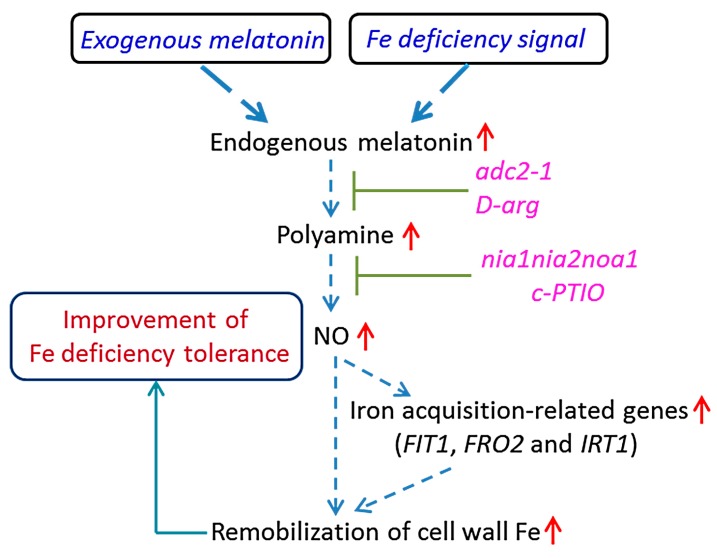
A proposed model illustrating the link between melatonin and improved tolerance of plants to Fe deficiency. The increased melatonin level induces the increment of the polyamine content, with the subsequent induction of NO accumulation. The enhancement of the NO signal then activates Fe deficiency responses and increases the remobilization cell wall Fe, which results in the increased soluble Fe in response to the low Fe supply. Dashed arrows indicate regulatory pathways. Red upright arrows denote increases in content or effects.

In conclusion, a model was proposed linking the melatonin-induced increase of polyamine levels to Fe deficiency responses (Figure 11). Here, we reported that Fe deficiency induced a remarkable increase of endogenous melatonin levels in *Arabidopsis* plants. Furthermore, exogenous application of melatonin greatly increased the remobilization of root cell wall Fe by the polyamine-mediated NO accumulation. Consequently, there was more soluble Fe in the roots and leaves, thereby alleviating the chlorosis caused by Fe deficiency. Thus, increased melatonin levels will be beneficial to reutilize Fe from the cell wall and, thus, confers more tolerance of plants to Fe-deficient conditions.

## Supplementary Materials

Supplementary materials can be found at www.mdpi.com/1422-0067/17/11/1777/s1.

## Abbreviations

NO	Nitric oxide
PTIO	2-(4-Carboxyphenyl)-4,4,5,5-tetramethylimidazoline-1–1-oxy-3-oxide
ADC	Arginine decarboxylase
Put	Putrescine
Spd	Spermidine
Spm	Spermine
