# Supplementary material for: Exogenous Melatonin Improves Plant Iron Deficiency Tolerance via Increased Accumulation of Polyamine-Mediated Nitric Oxide
Source: Int J Mol Sci. 2016 Oct 25;17(11):1777. doi: 10.3390/ijms17111777 (PMC5133778; doi:10.3390/ijms17111777)
Supplement: Supplementary file 1 [file ijms-17-01777-s001.pdf]

Supplementary Material: Exogenous Melatonin Improves Plant Iron Deficiency Tolerance via Increased Accumulation of Polyamine-Mediated Nitric Oxide

Cheng Zhou, Zhi Liu, Lin Zhu, Zhongyou Ma, Jianfei Wang and Jian Zhu

Table S1. Primers used in this study.

Gene	Usage	Sequence
ADC1	qPCR	5′CGGTTCCTGCCATTGGTCTC<br>5′GAACAGAGATGTTCCCGGA
ADC2	qPCR	5′CACACAGCTTTGCAGTGACC<br>5′TACGGCATGTTGTGGAACGA
FIT1	qPCR	5′CCAACACCTGTCGATGACCT<br>5′TTCACCACCGGCTCTAACAC
FRO2	qPCR	5′GCTTTGGTTATGGTGTGCGG<br>5′TCCCAGCTTTTGCTTCATCTC
IRT1	qPCR	5′TCTTGAAGAGAACCCGTGGC<br>5′GGTAACATCATTTGCGGGGC
ACTIN2	qPCR	5′GAAATCACAGCACTTGCA<br>5′AGCCTTTGATCTTGAGAG

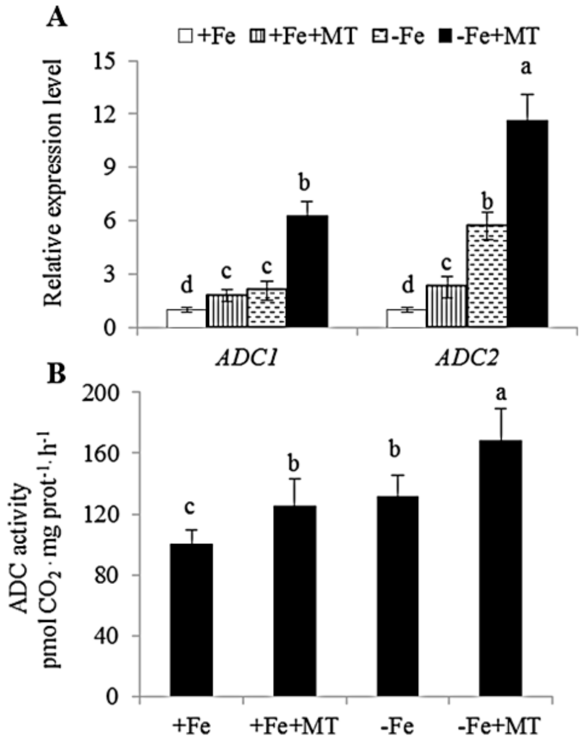

Figure S1. Effects of exogenous melatonin on the expression of ADC biosynthetic genes and the activities of ADC in *Arabidopsis* plants. 7-day-old seedlings were treated with or without 5 μM melatonin under −Fe and +Fe conditions for 8 day. These plants were then sampled to analyze the transcription levels of *ADC1* and *ADC2* (**A**), and the activities of ADC (**B**). Each bar is the mean ± SE of at least three replicates, and different lowercase letters above the bars indicate significant difference using Tukey's test at $p < 0.05$.
